# Patellofemoral alignment and geometry and early signs of osteoarthritis are associated in patellofemoral pain population

**DOI:** 10.1111/sms.13641

**Published:** 2020-03-18

**Authors:** Joost F. A. Eijkenboom, Rianne A. van der Heijden, Janneke L. M. de Kanter, Edwin H. Oei, Sita M. A. Bierma‐Zeinstra, Marienke van Middelkoop

**Affiliations:** ^1^ Department of General Practice Erasmus MC University Medical Center Rotterdam The Netherlands; ^2^ Department of Radiology & Nuclear Medicine Erasmus MC University Medical Center Rotterdam The Netherlands

**Keywords:** alignment, cartilage, geometry, magnetic resonance imaging, osteoarthritis, patellofemoral joint, patellofemoral pain, perfusion, structural abnormalities

## Abstract

**Background:**

Patellofemoral pain (PFP) patients show increased prevalence of patellar malalignment. Structural and alignment abnormalities of the patellofemoral joint (PFJ) may play a role in development of PFP and patellofemoral osteoarthritis (PFOA).

**Objectives:**

Evaluating associations of patellofemoral alignment and femoral geometry with bony and cartilaginous abnormalities in PFP patients and healthy control subjects.

**Methods:**

Data from a case‐control study were used (64 PFP subjects, 70 control subjects, 57% female, age 23.2 (6.4)). Alignment and femoral geometry measures in the PFJ were determined using MRI. Structural abnormalities in the PFJ associated with OA (bone marrow lesions, osteophytes, minor cartilage defects and Hoffa‐synovitis), quantified cartilage composition (T1ρ relaxation times) in the PFJ and perfusion within the patellar bone were examined using different MRI techniques. Associations were analyzed using regression analyses, adjusted for potential confounders.

**Results:**

Lateral patellar tilt was negatively associated with presence of osteophytes on both patella (OR 0.91; 95% CI 0.84 to 0.98), anterior femur (OR 0.92; 95% CI 0.84 to 0.99) and minor cartilage defects on patella (OR 0.91; 95% CI 0.84 to 0.99). Patella alta was positively associated with the presence of bone marrow lesions in the patella and minor cartilage defects (OR 48.33; 95% CI 4.27 to 547.30 and OR 17.51; 95% CI 1.17 to 262.57, respectively). Patella alta and medial patellar translation were positively associated with T1ρ relaxation times within trochlear cartilage (β 5.2; 95% CI 0.77 to 9.58, and 0.36; 95% CI 0.08 to 0.64, respectively). None of the alignment and geometry measures were associated with bone perfusion.

**Conclusion:**

Our study implies that associations between patellofemoral alignment and geometry and structural joint abnormalities linked to OA are already present in both PFP patients and healthy control subjects.

## 1 INTRODUCTION

Patellofemoral pain (PFP) is a common knee complaint, especially in active adolescents and runners.^1^ Contrary to earlier beliefs, increasing evidence suggests that PFP is not a self‐limiting disease.^2^, ^3^ Different treatment strategies are applied in the treatment of PFP, including strengthening exercises, taping, and orthoses, but the reported effects are small to moderate.^4^, ^5^, ^6^ In order to optimize treatment outcomes, there is need to elucidate the pathogenesis of PFP. There is overall consensus that the pathogenesis of PFP is multifactorial: numerous factors associated with PFP have been reported in literature, but the interaction between these proposed risk factors and the clinical entity of PFP remains unclear.^7^

In a recent study, several novel magnetic resonance imaging (MRI) techniques were applied to gain more insight in the etiology of PFP.^8^ Although it was long believed that PFP was caused by chondromalacia,^9^ van der Heijden et al showed that structural abnormalities (eg, cartilage loss and osteophytes) seen on MRI are not associated with the presence of PFP.^8^, ^10^ Additionally, no differences in cartilage composition were seen between groups.^11^ However, in another study, significantly higher T1ρ relaxation times in lateral patellar cartilage were found in a group of maltracking (eg, patellar tilt present) PFP patients compared with healthy controls, indicating an impaired biochemical composition.^12^ A recently published study showed that features of radiographic and MRI‐defined patellofemoral osteoarthritis (PFOA) are evident in 20%‐30% of adults aged 26 to 50 years.^13^ This suggests that particular subgroups in both patient and control groups appear to have structural abnormalities such as cartilage lesions and osteophytes in the patellofemoral joint (PFJ) and may be at increased risk to develop OA later in life. PFP as a predisposing factor for PFOA has been hypothesized in multiple studies but strong evidence is lacking.^14^, ^15^, ^16^ This hypothesis is merely based on the converging risk factors of both diseases.^17^, ^18^, ^19^ Malalignment and maltracking are risk factors found in both PFP and PFOA populations.^20^, ^21^ Macri et al recently showed in a literature review that there is strong evidence for associations between trochlear morphology (sulcus angle and trochlear depth) and the presence of PFOA.^20^ In PFP patients, it has been shown that the distance between the tibial tuberosity and trochlear groove (TT‐TG) were larger in a subgroup of extreme lateral maltracking patients which indicates an association between maltracking and joint geometry.^22^ Other frequently reported measures of alignment, that is, patella alta and patellar tilt, have been associated with the presence of PFP^23^ but also with OA progression.^24^ This suggests that malalignment is an important risk factor for both PFP and PFOA and that particular PFP patients might show early signs of OA development.

Considering that maltracking subgroups have previously been found within PFP populations^25^ and maltracking has been associated with OA,^20^ it is hypothesized that malalignment in PFP is associated with structural damage in the PFJ. Therefore, the aim of the present study was to investigate the association between PFJ alignment and geometry measures, and structural changes in bone and cartilage in individuals with and without PFP.

## 2 METHODS

### 2.1 Study population

For current study purpose, data from a previously conducted case‐control study were used in which subjects aged between 14 and 40 years with PFP and healthy control subjects were included.^8^, ^26^ PFP patients were included by their general practitioner, physiotherapist, or sports physician if they were diagnosed with PFP based on the presence of at least three of the following symptoms: crepitus or pain while stair climbing, squatting, running, cycling, or sitting for a prolonged period with the knee flexed. A minimum symptom duration of two months to a maximum of 2 years was required for the PFP patients. Patients were excluded if they currently had a defined pathological knee condition of the affected knee, previous surgery or injury of the affected knee, or previous episodes of PFP more than two years ago or if onset of PFP occurred after trauma. After inclusion by the healthcare professional, the inclusion criteria were checked by the researcher (RA), who checked the presence of possible exclusion diagnoses such as tendinopathy and meniscal lesions. Sports team members, friends, or colleagues of the PFP patients were included as control subjects, when possibly matched for age, body mass index (BMI), sex, and activity level. Control subjects were excluded on current or past PFP, traumatic injury or surgery on both knees or if they were first‐grade family members of patients. Contraindications for contrast‐enhanced MRI and insufficient knowledge of the Dutch language were exclusion criteria for all participants. The medical ethics committee of the Erasmus MC approved this study (protocol MEC‐2012‐342), informed consent was accordingly obtained from all participants and the rights of the subjects were protected before measurements took place.

### 2.2 Measurements

All study subjects completed a questionnaire including questions on demographics (gender, age, BMI), sports participation (yes or no, before onset of pain and during study), and knee complaints (duration of the complaints, paint at rest, and pain during activity using a numerical rating scale of 0‐10 and function, measured with the Anterior Knee Pain Scale (AKPS) on a 0‐100 scale). All subjects underwent 3 Tesla MRI (Discovery MR750, GE Healthcare) measurements using a dedicated 8‐channel knee coil (Invivo Inc). The most painful knee was chosen in patients, while a random knee was chosen if both were equally affected or in the case of control subjects.

MRI measurements included two 3D high‐resolution sequences (slice thickness 0.5 mm, 0 mm interslice gap): routine sagittal spoiled gradient echo (SPGR) sequences and routine sagittal fat‐saturated SPGR sequences. Furthermore, 3D fast spin echo (FSE) sagittal T1ρ mapping sequences were acquired (slice thickness 3 mm, no interslice gap). Due to ethical reasons**,** only the adult participants underwent dynamic contrast‐enhanced (DCE) MRI, performed with a sagittal fat suppressed 3D SPGR sequence (slice thickness 5mm, no interslice gap) with a temporal resolution of 10 seconds and lasting for 35 phases after intravenous contrast administration (0.2 mmol/kg Magnevist (Bayer)). The exact MRI protocol parameters can be found in Appendix S1.

### 2.3 MRI analysis

#### 2.3.1 Patellar alignment and femoral geometry

Common alignment (Insall‐Salvati ratio [ISR], patellar translation, patellar tilt) and femoral geometry (sulcus depth, sulcus angle, TT‐TG) measures of the PF joint were performed, as advised by the stepwise systematic approach of Chhabra et al^27^ (Table 1). The ISR was calculated by dividing patellar tendon length by the maximal diagonal length of the patella on the midline sagittal MRI image, a higher ratio indicates a higher riding patella (alta) and a lower ratio indicates a lower riding patella (baja). Patellar translation was measured as the distance between perpendicular lines drawn on an axial image from the medial edge of the patella through the most anterior point of the medial condyle. Positive values indicate lateral translation, and negative values indicate medial translation. Patellar tilt was defined as the angle between two lines drawn along the bony lateral patellar facet and the tangent to the anterior femoral condyles at the level of the patellar midpoint. Positive values indicate tilt in the lateral direction whereas negative values indicate tilt in the medial direction. Sulcus depth was defined as the distance of the deepest area of the trochlear groove relative to the mean of the femoral condyle outsets, and sulcus angle was defined as the angle between two lines from condyle outsets to the deepest area of the trochlear groove, measured on axial images. The TT‐TG distance was defined as the maximum distance between two lines drawn perpendicular to the deepest area of the trochlear groove and the center of the patellar tendon insertion on the tibial tuberosity on axial images.

**Table 1:** Alignment and geometry measures

Alignment and shape measure	Definition	Positive direction	Negative direction
ISR	Patellar tendon length divided by maximal diagonal length of patella	High riding patella (alta)	Low riding patella (baja)
Patellar translation	Patella is translated in lateral or medial direction compared to trochlea	Patella is translated in lateral direction	Patella is translated in medial direction
Patellar tilt	The patella is not parallel with the condyle outsets	Lateral tilt (lateral side of patella is closer to trochlea)	Medial tilt (medial side of patella is closer to trochlea)
Sulcus depth	Depth of sulcus groove compared to femoral condyle outsets	—	—
Sulcus angle	Angle between the condyle outsets	—	—
TT‐TG	Distance between tibial tuberosity and trochlear groove in the axial plane (requires multiple MRI slices)	—	—

Note

Abbreviations: ISR, Insall‐Salvati ratio; TT‐TG, tibial tuberosity trochlear groove distance.

#### 2.3.2 Structural abnormalities

**Figure 1:**
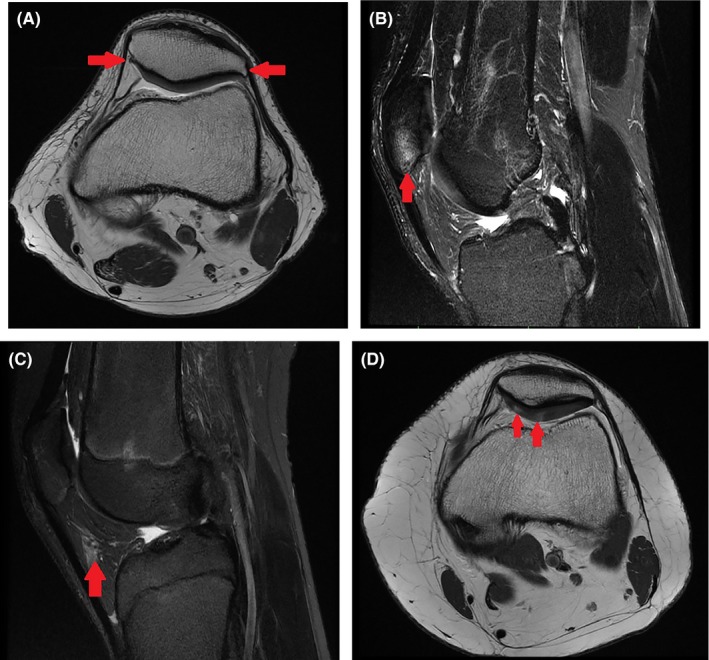
Structural abnormalities. A, Osteophytes on patella (axial proton density weighted MRI); B, Bone marrow lesions in patella (sagittal T2‐weighted fat‐saturated MRI); C, Hoffa‐synovitis (sagittal T2‐weighted fat‐saturated MRI); D, Minor cartilage defects in patellar cartilage (axial proton density weighted MRI). Arrows highlight affected areas

The high‐resolution MRI and routine clinical MRI scans were used to score structural abnormalities within the PF joint, using the semi‐quantitative magnetic resonance imaging osteoarthritis knee score (MOAKS). For the present study, MOAKS scores of the most prevalent^8^ and relevant features were selected and dichotomized (0 = not present, ≥1 = present): Osteophytes on patella, osteophytes on anterior femur, bone marrow lesions (BMLs) in patella, BMLs in anterior femur, Hoffa‐synovitis and minor cartilage defects on patella (Figure 1). Hoffa‐synovitis was defined as high signal intensity of the superolateral part of the Hoffa fat pad, as described by Chhabra et al^27^ Minor cartilage defects were defined as high signal intensity, hypertrophy, fraying or fissuring of the cartilage. Alignment and geometry measurements and scoring of MOAKS features were performed by a senior resident (JLd.K) in radiology with musculo‐skeletal sub‐specialization. All findings were verified by an experienced musculo‐skeletal radiologist (E.O). Both readers were blinded for participant status.^8^

#### 2.3.3 Cartilage composition and bone perfusion

T1ρ mapping MRI (3D fast spin echo sequence with 5 different spin lock times and a spin lock frequency of 500 Hz) was used to quantitatively assess cartilage quality in which higher T1ρ relaxation time values are assumed to indicate less glycosaminoglycan.^28^ Detailed methods have been described elsewhere.^11^ For analyses, weighted mean relaxation times were obtained separately for femur and patellar cartilage. DCE‐MRI scans were used to calculate Ktrans and Kep measures of blood perfusion in the patellar bone, as described by van der Heijden et al.^29^, ^30^ Weighted mean of Ktrans and Kep in the whole patella was calculated for analysis.

### 2.4 Statistical analysis

Statistical analyses were performed in SPSS 21 (SPSS Inc). Descriptive statistics were used to describe subject characteristics, structural abnormalities, cartilage composition, perfusion parameters, and alignment and geometry measures. Associations between alignment and geometry measures (independent variables) and cartilage composition, and perfusion and structural abnormalities (dependent variables) were analyzed using multivariate linear and logistic regression techniques for each alignment or geometry measure separately, and adjustments were made for age, sex, BMI, and the presence of PFP (case/control status). Linear regression results were presented by unstandardized regression coefficients (β) with 95% confidence intervals while logistic regression results were presented by odds ratios (OR) with 95% confidence intervals. Missing values were handled by performing complete case analysis.

## 3 RESULTS

The study population consisted of 76 females (56.7%) and 58 males (43.3%), with a mean age of 23.2 (6.4) years and BMI of 22.8 (3.4). Perfusion parameters were available for 35 PFP patients and 44 control subjects (adult subjects only), T1ρ relaxation times were available for 42 PFP patients and 50 control subjects (T1ρ sequence was unavailable at start of study) and structural abnormalities were available for all 64 PFP patients and 70 control subjects. Subject characteristics are presented in Table 2. Alignment and femoral geometry measures were available for all subjects (Table 3). No differences in alignment and femoral geometry measures were observed between groups.

**Table 2:** Characteristics of study participants

	PFP n = 64	Control n = 70
Female gender (n (%))	35 (54.7)	41 (58.6)
Age (mean (SD))	23.4 (7.0)	23.1 (5.9)
BMI (mean (SD))	23.6 (3.8)	22.3 (3.0)
Presence of crepitation (n (%))	29 (45.3)	20 (29.0)
Sport participation		
During inclusion (n (%))	38 (40.6)	55 (78.6)
Before onset of pain (n (%))	48 (85.7)	NA
Pain in rest, NRS/10 (mean (SD))	3.9 (2.5)	NA
Pain during exercise, NRS/10 (mean (SD))	6.6 (2.2)	NA
AKPS score, 0‐100 (mean (SD))	66.3 (11.6)	NA
Complaint duration, months (mean, (SD))	12.0 (7.1)	NA
Bilateral complains	33 (51.6)	NA

Patellar bone perfusion (Mean (SD))	n = 35	n = 44
Log Mean K_trans_ (min^−1^)	−1.89 (0.31)	−1.98 (0.29)
Log Mean K_ep_ (min^−1^)	−0.49 (0.22)	−0.58 (0.37)

Cartilage Composition (Mean (SD))	n = 42	n = 50
Mean T1ρ femur cartilage (ms)	661.6 (63.8)	659.8 (66.2)
Mean T1ρ patella cartilage (ms)	657.8 (83.7)	669.4 (55.1)

Structural abnormalities (n (%))	n = 64	n = 70
Osteophytes patella	45 (70.3)	42 (60.0)
Osteophytes anterior femur	12 (18.8)	9 (12.9)
BMLs patella	34 (53.1)	36 (51.4)
BMLs anterior femur	5 (7.8)	8 (11.4)
Meniscus extrusion	10 (15.6)	9 (12.9)
Hoffa‐synovitis	29 (45.3)	27 (38.6)
Minor cartilage defects patella	15 (23.4)	15 (21.4)

Abbreviations: AKPS, anterior knee pain score; BMI, body mass index; BMLs, bone marrow lesions; Kep, rate constant back from tissue compartment to vascular space; Ktrans, volume transfer constant from vascular space into tissue compartment; NRS, numerical pain scale; PFP, patellofemoral pain.

**Table 3:** PFJ alignment and geometry measures (Means (SD)) of patients and healthy control subjects

	PFP n = 64	Control n = 70
ISR	1.2 (0.2)	1.2 (0.2)
Patellar translation^a^, mm	0.3 (2.6)	0.4 (2.3)
Patellar tilt^a^, °	8.3 (6.0)	10.1 (5.4)
Sulcus depth, mm	6.1 (1.0)	6.2 (1.0)
Sulcus Angle, °	137.3 (4.9)	136 (4.7)
TT‐TG distance, mm	12.4 (4.4)	12.3 (4.2)

Abbreviations: ISR, Insall‐Salvati ratio; PFP, patellofemoral pain; TT‐TG, tibial tuberosity trochlear groove distance.

^a^ Positive values are in lateral direction; negative values are in medial direction.

Table 4 presents the association between alignment and geometry measures and MRI outcomes. Lateral patellar tilt (°) was negatively associated with the presence of osteophytes on both patella and anterior femur (OR 0.91; 95% CI 0.84 to 0.98 and OR 0.92; 95% CI 0.84 to 0.99, respectively) and minor cartilage defects on patella (OR 0.91; 95% CI 0.84 to 0.99). In addition, sulcus angle (°) was negatively associated with the presence of Hoffa‐synovitis (OR 0.91; 95% CI 0.84 to 0.99) and lateral patellar tilt (°) was positively associated with the presence of Hoffa‐synovitis (OR 1.08; 95% CI 1.01 to 1.16). ISR was positively associated with the presence of BMLs in the patella and minor cartilage defects on the patella (OR 48.33; 95% CI 4.27 to 547.30 and OR 17.51; 95% CI 1.17 to 262.57, respectively). Sulcus depth (mm) was negatively associated with presence of BMLs in the anterior femur (OR 0.45; 95% CI 0.22 to 0.93).

**Table 4:** Associations between alignment and geometry measures and MRI outcome measures

	ISR	Patellar translation^a^ (mm)	Patellar tilt^a^ (°)	sulcus depth (mm)	sulcus angle (°)	patellar TT‐TG (mm)
	OR (95% CI)	OR (95% CI)	OR (95% CI)	OR (95% CI)	OR (95% CI)	OR (95% CI)
Osteophytes patella	5.00 (0.43;57.80)	1.12 (0.95;1.32)	**0.91 (0.84;0.98)**	1.17 (0.76;1.78)	0.96 (0.89;1.04)	1.04 (0.95;1.14)
Osteophytes anterior femur	1.12 (0.06;19.57)	1.05 (0.87;1.27)	**0.92 (0.84;0.99)**	1.15 (0.68;1.95)	1.09 (0.98;1.22)	1.03 (0.92;1.15)
BMLs patella	**48.33 (4.27;547.30)**	0.91 (0.78;1.06)	0.97 (0.91;1.04)	0.88 (0.59;1.30)	1.07 (0.99;1.15)	1.05 (0.96;1.14)
BMLs anterior femur	3.89 (0.11;142.28)	0.95 (0.75;1.21)	0.99 (0.89;1.11)	**0.45 (0.22;0.93)**	1.06 (0.93;1.20)	1.02 (0.88;1.17)
Hoffa‐synovitis	1.47 (0.16;1.45)	0.90 (0.77;1.05)	**1.08 (1.01;1.16)**	1.04 (0.69;1.55)	**0.91 (0.84;0.99)**	1.02 (0.94;1.11)
Minor cartilage defects patella	**17.51 (1.17;262.57)**	1.11 (0.93;1.33)	**0.91 (0.84;0.99)**	0.93 (0.58;1.49)	1.06 (0.96;1.16)	1.04 (0.95;1.15)

	β (95% CI)	β (95% CI)	β (95% CI)	β (95% CI)	β (95% CI)	β (95% CI)
T1ρ femur	**5.18 (0.77;9.58)**	−**0.36 (−0.64;** −**0.08)**	−0.04 (−0.16;0.08)	0.65 (−0.14;1.44)	0.01 (−0.14;0.17)	−0.02 (−0.19;0.14)
T1ρ patella	2.30 (−2.61;7.21)	−0.20 (−0.51;0.11)	−0.06 (−0.19;0.08)	0.04 (−0.83;0.92)	0.01 (−0.16;0.18)	−0.07 (−0.25;0.11)
Log mean Kep	−0.41 (−1.37;0.56)	0.04 (−0.02;0.09)	0.00 (−0.03;0.02)	−0.09 (−0.25;0.08)	0.01 (−0.03;0.04)	−0.02 (−0.05;0.02)
Log mean Ktrans	−0.24 (−0.69;0.20)	0.01 (−0.02;0.04)	0.00 (−0.01;0.01)	−0.01 (−0.09;0.06)	0.00 (−0.02;0.01)	0.01 (−0.02;0.01)

Note

Odds ratios (95% CI) unless stated otherwise, adjusted for age, sex, BMI, and presence of PFP. Significant associations in bold.

Abbreviations: BMLs, bone marrow lesions; ISR, Insall‐Salvati ratio; Kep, rate constant back from tissue compartment to vascular space; Ktrans, volume transfer constant from vascular space into tissue compartment; PFP, patellofemoral pain; TT‐TG, tibial tuberosity trochlear groove distance.

^a^ Positive values are in lateral direction, and negative values are in medial direction.

Insall‐Salvati ratio was positively associated with T1ρ relaxation times of the trochlear cartilage (β 5.2; 95% CI 0.77 to 9.58) while lateral patellar translation was negatively associated with T1ρ relaxation times of the trochlear cartilage (β −0.36; 95% CI −0.64 to −0.08, respectively). None of the alignment or geometry measures were associated with bone perfusion.

## 4 DISCUSSION

The purpose of this study was to evaluate the associations between PFJ alignment and femoral geometry measures, and changes in bone and cartilage associated with OA. The results demonstrate that, regardless of the presence of PFP, several alignment and geometry measures already seem to be associated with cartilage and bone abnormalities at a young age. Patella alta, patellar tilt, and trochlear geometry were associated with the presence of osteophytes and BMLs on both the anterior femur and patella, as well as Hoffa‐synovitis and minor cartilage defects on the patella. Additionally, patella alta and medial translation were associated with higher T1ρ relaxation times in the trochlear cartilage, indicating altered biochemical cartilage composition. In contrast to our hypotheses, no alignment or geometry measure was associated with bone perfusion.

Although our study provides no causality, one can speculate why some of these associations exist. Patella alta can result in a delayed engagement of the patella with the trochlea during flexion and may therefore reduce contact area during early flexion.^31^ The increase of shear stress as a result of reduction in contact area may result in bone marrow lesions and cartilage damage. Additionally, abnormal patellar positioning or sulcus geometry can also be hypothesized to reduce contact area in the PFJ during movement, resulting in higher shear stresses and structural damage.

Aberrant PFJ kinematics have previously been associated with malalignment^25^ and abnormal joint geometry.^32^ These abnormal kinematics are suggested to impact contact areas in the joint^33^ and lead to increased joint stress in the PFJ,^34^ which in turn have been hypothesized to induce loss of cartilage and onset of BMLs^35^ and structural damage.^19^ The findings of this study support these theories, as we found multiple measures of alignment that correlate to the presence of BMLs, osteophytes, and minor cartilage defects. Although there is limited evidence of the presence of greater shear stresses in PFP patients,^34^ the association between malalignment, joint stresses, and OA progression has been shown in knee valgus and varus patients.^36^ Therefore, future research should investigate whether these alignment and shape measures in PFP patients indeed result in increased shear stresses in the PFJ and consequently result in early signs of OA. This in order to get a better insight into potential subgroups of PFP patients that are at high risk to develop OA at later age.

The associations between abnormal alignment and femoral geometry and the presence of structural abnormalities found in the present study were equal in both PFP patients and healthy control subjects. Hence, there seems to be both PFP patients and healthy control subjects with particular bone alignment and geometry characteristics who have an increased prevalence of osteophytes, BMLs, Hoffa‐synovitis, minor patellar cartilage defects, and deteriorated biochemical composition in femoral cartilage. This may indicate that a subgroup with specific alterations in alignment and bone geometry may be at greater risk to develop OA. It is so far unclear if these structural abnormalities will also result in definite OA. Further research into this young group with structural abnormalities could help understand the potential link between alignment and geometry and the development OA.

Many studies have already demonstrated an association between patellofemoral alignment and geometry and the presence of OA.^19^, ^20^ Those studies showed that patellar height, tilt, and shallow trochleae were associated with a higher prevalence of BMLs and other signs of OA in the PF joint.^19^, ^20^ A flattened trochlea could increase the chance of a laterally displaced patella, causing damage in the PFJ.^37^ Moreover, a recent multicenter study showed that patella alta is associated with worsening of structural features of PFOA.^38^ It has been theorized that a higher riding patella increases the maximal patellofemoral contact force and contact pressure by increasing the flexion needed for PFJ contact.^39^ While in the present study only a significant association with the presence of BMLs and minor cartilage defects on the patella was found, an identical trend was seen for all structural features assessed in this study. To further understand the influence of aberrant patellofemoral alignment and geometry on the later development of OA in a population with PFP, a longitudinal study with a long follow‐up is needed. While clinical implications have to be interpreted with caution, one could hypothesize that interventions influencing patellofemoral alignment, like bracing, could potentially prevent or delay the onset of OA in this subgroup.

A high riding or medially translated patella was associated with increased T1ρ relaxation times in femoral cartilage. The association between patella alta and the deterioration of the biochemical composition of cartilage may be explained by the increased contact forces in the PFJ, caused by patella alta.^39^ This is in line with other studies which have described a loss of cartilage thickness associated with knee malalignment.^40^ Wang et al found higher T1ρ values on the medial anterior femur in OA patients with knee varus.^41^ We suspected lateral and medial patellar translation to have a greater association with lateral and medial trochlear cartilage T1ρ values, respectively. However, additional explorative analyses in the current study with separate medial and lateral trochlear T1ρ relaxation times did not show a difference in the association with patellar translation between the lateral and medial compartment. Nonetheless, we did find a strong association between patella alta and impaired cartilage composition in the lateral trochlea.

No associations were found between patellar perfusion and alignment and geometry measures in our study. This indicates that the observed large perfusion variance between PFP patients in van der Heijden et al^30^ is probably not a result of differences in alignment or geometry measures between patients.

All conducted analyses were adjusted for age, sex, BMI, and the presence of PFP (case/control status). These adjustments consistently revealed that a higher age results in a higher prevalence of osteophytes, independent of the alignment, or geometry measure. This seems to be in line with literature on older OA subjects in which the prevalence of osteophytes, and consequently OA, increases with age.^42^ Yet, we also found higher age is associated with a decrease in the presence of BMLs. This seems to be in contrast with literature describing a higher prevalence in BMLs with increasing age.^43^ Why BMLs in our study populations seems to decrease with age is unclear. These associations were found in the total study population, but remained present in the separate control and PFP study subgroups.

### 4.1 Strengths and limitations

This is the first study investigating the associations between alignment and geometry in the PFJ and abnormalities in bone and cartilage associated with OA in a young population. For the analyses of the present study, continuous measures of joint alignment and femoral geometry were used to preserve statistical power, whereas clinical cutoff values for normal and abnormal alignment and geometry measures have been proposed in literature.^27^ We therefore exploratively tested these dichotomized variables in the current study and found similar directions of associations, although not all remained statistically significant, which is likely due to a loss of statistical power.^44^

A relatively large number of statistical tests were used within our relatively small population, possibly causing false‐positive findings (Type 1 error). Additionally, the small number of participants used for analysis resulted in large confidence intervals for some of our analyses, limiting the precision of the association. Because a clear hypothesis was formed based on earlier literature showing associations between PFJ kinematics and structural abnormalities, we think that the study findings add unique information about the etiology of PFP and the value of joint kinematics and structural abnormalities.

No reliability data are available from the alignment, geometry, and MOAKS measurements, and we can therefore not exclude intra‐observer deviations in our data. However, all measurements were performed by experienced blinded radiologists.

## 5 PERSPECTIVE

Our study showed no differences in alignment and femoral geometry measures between PFP and control groups. However, associations were found between alignment and geometry measures and structural joint abnormalities linked to OA in the PFJ in a young population, including PFP patients. This is in line with results found by Kang et al^45^ showing an increase in MRI abnormalities with changes in sulcus geometry. Since kinematics can be changed with noninvasive treatments like bracing^46^ and training,^47^ one could potentially delay or prevent the onset of PFOA in young people with malalignment in the PFJ. Further prospective studies are needed to investigate the exact relation between malalignment and early OA in young populations.

## ETHICAL APPROVAL

The medical ethics committee of the Erasmus MC approved this study (protocol MEC‐2012‐342). The authors certify that they have no affiliations with or financial involvement in any organization or entity with a direct financial interest in the subject matter or materials discussed in the article.

## Supporting information

Supplementary Material
