# Supplementary material for: Patellofemoral alignment and geometry and early signs of osteoarthritis are associated in patellofemoral pain population
Source: Scand J Med Sci Sports. 2020 Mar 18;30(5):885–93. doi: 10.1111/sms.13641 (PMC7187437; doi:10.1111/sms.13641)
Supplement: Supplementary file 1 — Supplementary Material [file SMS-30-885-s001.docx]

**Appendix Table 1:** MRI protocol parameters

| Sequence | 3D SPGR | T1_ρ_  mapping | DCE |
| --- | --- | --- | --- |
| Plane | Sagittal | Sagittal | Sagitall |
| Imaging mode | 3D | 3D | 3D |
| Sequence | SPGR | FSE | SPGR |
| Matrix (frequency) | 512 | 288 | 256 |
| Matrix (phase) | 512 | 192 | 128 |
| Number of slices | 216 | 36 | 14 |
| FOV (mm) | 150 | 150 | 380 |
| Slice thickness/gap (mm/mm) | 0.5/0.0 | 3.0/0.0 | 5.0/0.0 |
| TSL (ms) † | n.a. | 1 / 16 / 32 / 64 / 125 | n.a. |
| TE (ms) | 5.4 | n.a. | 1.7 |
| Flip angle (˚) | 12 | 90 | 30 |
| Repetition time (ms) | 17 | 1261 | 9.3 |
| Number of excitations | 0.75 | 0.5 | 0.66 |
| Fat saturated | Yes and No | Yes | Yes |
| Acquisition time (min) | 05:37 | 05:43 | ±6:00 |

n.a.: not applicable; SPGR: spoiled gradient-echo; FOV: field of view; TSL: spin lock time; TE: echo time, DCE: dynamic contrast enhanced

† spinlock frequency of 500Hz
